# Effects of Inspiratory Muscle Training in People with Chronic Obstructive Pulmonary Disease: A Systematic Review and Meta-Analysis

**DOI:** 10.3390/life14111470

**Published:** 2024-11-12

**Authors:** Bing Han, Zhuying Chen, Bing Ruan, Yongjie Chen, Yuanyuan Lv, Cui Li, Laikang Yu

**Affiliations:** 1 Key Laboratory of Physical Fitness and Exercise, Ministry of Education, Beijing Sport University, Beijing 100084, China; 13520753687@163.com (B.H.); sunflowerlyy@bsu.edu.cn (Y.L.); 2 Department of Strength and Conditioning Assessment and Monitoring, Beijing Sport University, Beijing 100084, China; zhuying20232120126@126.com (Z.C.);; 3 School of Sport Medicine and Rehabilitation, Beijing Sport University, Beijing 100084, China; ruanbing@bsu.edu.cn; 4 China Institute of Sport and Health Science, Beijing Sport University, Beijing 100084, China; 5 School of Physical Education (Main Campus), Zhengzhou University, Zhengzhou 450001, China; lc@zzu.edu.cn

**Keywords:** inspiratory muscle training, chronic obstructive pulmonary disease, inspiratory muscle strength, dyspnea, quality of life

## Abstract

This study aimed to investigate the effects of inspiratory muscle training (IMT) on inspiratory muscle strength, dyspnea, and quality of life (QOL) in COPD patients. A comprehensive search was undertaken on the Web of Science, Scopus, Embase, Cochrane, and PubMed databases, encompassing data published up to 31 March 2024. A meta-analysis was subsequently conducted to quantify the standardized mean difference (SMD) and 95% confidence interval (CI) for the effects of IMT in COPD patients. Sixteen studies met the inclusion criteria. IMT significantly improved inspiratory muscle strength (SMD, 0.86, *p* < 0.00001), dyspnea (SMD = −0.50, *p* < 0.00001), and QOL (SMD = 0.48, *p* = 0.0006). Subgroup analysis showed that <60% maximal inspiratory muscle pressure (PImax) IMT (inspiratory muscle strength, SMD = 1.22, *p* = 0.005; dyspnea, SMD = −0.92, *p* < 0.0001), IMT conducted for ≤20 min (inspiratory muscle strength, SMD = 0.97, *p* = 0.008; dyspnea, SMD = −0.63, *p* = 0.007; QOL, SMD = 1.66, *p* = 0.007), and IMT conducted >3 times per week (inspiratory muscle strength, SMD = 1.06, *p* < 0.00001; dyspnea, SMD = −0.54, *p* < 0.00001; QOL, SMD = 0.48, *p* = 0.0009) had greater effects. This meta-analysis provides clinicians with evidence supporting the recommendation that COPD patients engage in IMT at <60% PImax for more than 3 times per week, with each session lasting no more than 20 min, to improve inspiratory muscle strength, dyspnea, and QOL.

## 1. Introduction

Chronic obstructive pulmonary disease (COPD) is a preventable and treatable disease characterized by persistent, often progressive airflow obstruction due to abnormalities in the airways (bronchitis, bronchiolitis) and/or alveoli (emphysema) which leads to chronic respiratory symptoms such as dyspnea, cough, sputum, and/or aggravation [1]. COPD symptoms significantly impact a person’s activity, health status, and quality of life (QOL), particularly as dyspnea is a major contributor to COPD-related anxiety and disability. These symptoms can also affect family life and the individual’s ability to perform daily activities, like household chores and climbing stairs. Apart from pulmonary symptoms, COPD may also manifest with systemic symptoms, including fatigue, weight loss, and sleep disturbances, as well as psychiatric symptoms like depression and anxiety, which severely compromise QOL [2]. Studies have shown that varying degrees of COPD are associated with reduced inspiratory muscle function [3]. Patients with COPD experience a decline in the functional strength of inspiratory muscles, particular those with moderate to severe COPD, where muscle strength typically falls between 40% and 60% of the predicted normal value, with varying degrees of decline. This deterioration can stem from a multitude of factors, including lung hyperinflation, muscle mass loss, frequent exacerbations of respiratory infection symptoms (once or more times a year), increased resting energy expenditure, corticosteroid use, individual muscle strength weakness, and aging [3,4].

As COPD progresses, the obstruction of airflow is accompanied by chest hyperinflation, which mechanically disadvantages the inspiratory muscles in terms of the length-tension relationship. The diaphragm becomes shortened and flattened, reducing its resting length and maximum tension-generating capacity [5]. This diminishes the diaphragm’s ability to exert force, weakening the inspiratory muscles’ function, evident in a decrease in maximal inspiratory muscle pressure (PImax). The extent to which hyperinflation contributes to the decline in inspiratory muscle strength remains unclear, but even minor increases in hyperinflation can significantly reduce PImax [3]. Consequently, reduced inspiratory muscle strength exacerbates dyspnea and puts individuals at risk of respiratory muscle fatigue. In addition, intensified breathing difficulties can limit physical activity, significantly affecting QOL.

At present, non-drug therapy constitutes an essential component of the comprehensive treatment of COPD. Inspiratory muscle training (IMT) has been extensively employed in both healthy individuals and those with illnesses, particularly the elderly, and its effects have been proven to positively influence not only inspiratory muscle strength but also dyspnea, exercise capacity, QOL, and various other health parameters [6]. A recent meta-analysis showed significant improvements in balance among both healthy participants and participants with diseases following IMT treatment, though the effect on functional activity remained inconclusive [6]. In addition, Bissett et al. [7] demonstrated that IMT is a safe and viable option for ventilator-dependent intensive care unit (ICU) patients, potentially enhancing weaning and improving inspiratory muscle strength and QOL. Furthermore, Ferraro et al. [8] conducted an 8-week unsupervised family-based IMT program for healthy elderly individuals, resulting in significant improvements in inspiratory muscle strength. Moreover, Figueiredo et al. [9] demonstrated that IMT can improve the strength and endurance of the respiratory muscles, thereby alleviating feelings of respiratory fatigue and dyspnea, significantly improving respiratory muscle function.

Numerous studies have demonstrated that IMT exerts a positive influence on COPD patients, but the specific advantages and ultimate conclusions remain inconclusive. Lötters F et al. [10] evaluated the effectiveness of IMT in COPD patients and concluded that IMT serves as a vital adjunct to pulmonary rehabilitation programs for COPD patients. Nevertheless, the assessment of IMT’s impact on inspiratory muscle strength and QOL was found to be imprecise. Additionally, Crowe et al. [11] conducted a meta-analysis on the effects of IMT and alternative interventions in adult COPD patients, reinforcing the notion that IMT enhances improve inspiratory muscle strength and endurance. Furthermore, in the meta-analyses by O’Brien et al. [12] and Beaumont et al. [13], IMT or a combination of IMT with exercise/pulmonary rehabilitation (PR) was compared to other rehabilitation interventions in adult COPD patients. Their findings indicated that combining IMT with exercise may significantly improve inspiratory muscle strength and exercise tolerance outcomes in COPD patients. The use of threshold devices for IMT improved inspiratory muscle strength, exercise capacity, and QOL while mitigating dyspnea. Nevertheless, when compared to PR alone, IMT did not confer additional benefits in alleviating breathing difficulties during PR. Moreover, a recent meta-analysis evaluating the effectiveness of various breathing exercises in COPD patients revealed that IMT was solely beneficial for dyspnea relief, without notable improvements in inspiratory muscle strength or QOL [14]. Notably, in the aforementioned study, the control groups received interventions other than IMT, impeding an accurate and holistic assessment of IMT’s effects on COPD patients. However, in the control groups of the studies we included, apart from usual care and sham IMT, all other IMT interventions were of low intensity and did not involve any additional interventions. Notably, we conducted subgroup analyses based on session time, frequency, and intensity, which can provide an optimal exercise prescription for COPD patients.

Therefore, we conducted a comprehensive systematic review and meta-analysis of randomized controlled trials (RCTs) to investigate the effects of IMT on inspiratory muscle strength, dyspnea, and QOL in COPD patients.

## 2. Materials and Methods

### 2.1. Design

This systematic review was conducted following the Preferred Reporting Items for Systematic Evaluation and Meta-Analyses (PRISMA, 2020) guidelines [15]. The protocol is registered with PROSPERO (CRD42024554445).

### 2.2. Search Strategy

We searched the Web of Science, Scopus, Embase, Cochrane, and PubMed databases from inception to 31 March 2024, for RCTs using the following keywords and MESH terms: inspiratory muscle training and chronic obstructive pulmonary disease (Supplementary Materials). The screening and selection procedure was autonomously carried out by two authors (BH and ZC). In instances of disagreement, a third author (LY) was engaged in the deliberation, promoting a collaborative effort until a unanimous decision was achieved.

### 2.3. Eligibility Criteria

The inclusion criteria were as follows: (1) studies adopting a RCT design; (2) involving participants diagnosed with COPD; (3) containing both an intervention group and a control group; and (4) using inspiratory muscle strength, dyspnea, or QOL as the outcome measures.

The excluded criteria were as follows: (1) publications not in English; (2) conference articles; (3) review articles; (4) studies including outcome indicators that could not be transformed into mean and standard deviation (SD); and (5) research lacking a control group.

### 2.4. Data Extraction

Utilizing a standardized data extraction form, two authors (BH and ZC) independently extracted the data. In cases where discrepancies arose in extracted data, they collaboratively conducted a second round of extraction to validate the accuracy of the information. The following data and information were extracted: characteristics of the included studies (the primary author’s surname, publication year), sample size, participant characteristics (age, sex, COPD prevalence), exercise protocols (type, intensity, intervention duration, frequency, session duration), and the mean values with SD of the key outcomes.

### 2.5. Methodological Quality Assessment

To evaluate the methodological quality of the included studies, we employed the Cochrane risk of bias tool (RoB2) [16,17]. The RoB2 includes seven key domains: random sequence generation (selection bias), allocation concealment (selection bias), blinding of participants and personnel (performance bias), blinding of outcome assessment (detection bias), incomplete outcome data (attrition bias), selective reporting (reporting bias), and other biases [18]. Each study was appraised along these dimensions, categorizing the risk as “low”, “uncertain”, or “high”. Two authors (BH and ZC) independently assessed the methodological quality of the included studies, and any discrepancies were resolved through the involvement of a third author (LY).

### 2.6. Statistical Analysis

For the purpose of meta-analysis, the mean values accompanied by their SD were extracted from each included study. In instances where the research data were presented as standard error (SEM), a conversion was made to SD [19,20]. To synthesize the data, a random-effects model was used in this study, yielding standardized mean differences (SMDs) and 95% confidence intervals (CIs). The extent of heterogeneity among the included studies was evaluated using *I*^2^, with interpretations as follows: *I*^2^ < 25% indicating no heterogeneity, 25% to 50% indicating low heterogeneity, 50% to 75% indicating moderate heterogeneity, and *I*^2^ > 75% indicating high heterogeneity [21,22]. Forest plots were generated using RevMan 5.4 software and funnel plots were generated using Stata 17 software, with statistical significance set at *p* < 0.05 for all results.

## 3. Results

### 3.1. Study Selection

**Figure 1:**
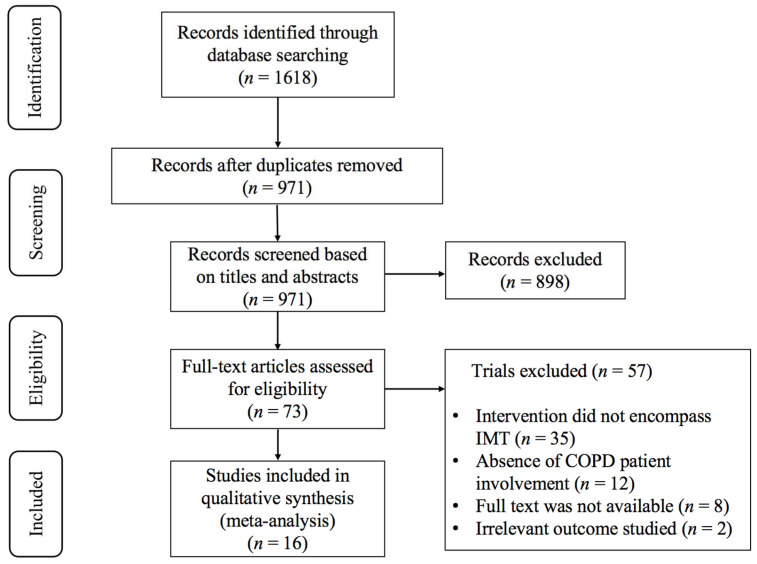
PRISMA flowchart of study selection.

As shown in Figure 1, the collaborative efforts of two authors yielded a total of 1618 retrieval records. Following the elimination of duplicate studies, the pool was narrowed down to 971 studies. Subsequently, a rigorous screening process involving the examination of titles and abstracts led to the exclusion of an additional 898 studies that did not meet the inclusion criteria. After a meticulous full-text assessment, 57 studies were disqualified based on several criteria: (1) the intervention not encompassing IMT (*n* = 35); (2) an absence of COPD patient involvement (*n* = 12); (3) the full text not being accessible for review (*n* = 8); (4) the outcomes studied being deemed irrelevant to the research objectives (*n* = 2). Finally, 16 studies [23,24,25,26,27,28,29,30,31,32,33,34,35,36,37,38] met the inclusion criteria and were selected for further analysis.

### 3.2. Characteristics of the Included Studies

Table S1 summarizes the included studies’ composition, encompassing 700 participants in the intervention group and 644 participants in the control group, all diagnosed with COPD at the GOLD Ⅱ–Ⅳ stages. The studies accommodated multiple intervention groups within a single study, while the control groups were subjected to either a placebo intervention or no additional intervention beyond their daily routines, medications, and usual care. The intervention duration varied significantly across the studies, ranging from as brief as 3 weeks to as extensive as 15 months, with session duration lasting from 3 min to 60 min. Notably, 10 studies provided data on inspiratory muscle strength [23,24,25,26,28,29,32,36,37,38], primarily measured using PImax. Additionally, 11 studies provided data on dyspnea [24,25,27,28,30,31,32,33,35,36,38], primarily assessed using the baseline dyspnea index/transition dyspnea index (BDI/TDI), modified Medical Research Council (mMRC) scale, Borg scale, and San Diego Shortness of Breath Questionnaire (SOBQ). Furthermore, seven studies provided data on QOL [26,27,31,32,33,34,38], primarily evaluated using the Chronic Respiratory Disease Questionnaire (CRDQ), St. George’s Respiratory Questionnaire (SGRQ), Short Form 36-Item Health Survey (SF-36), Chronic Airway Test (CAT), and Clinical COPD Questionnaire (CCQ).

### 3.3. Meta-Analysis

#### 3.3.1. Effects of IMT on Inspiratory Muscle Strength in COPD Patients

**Figure 2:**
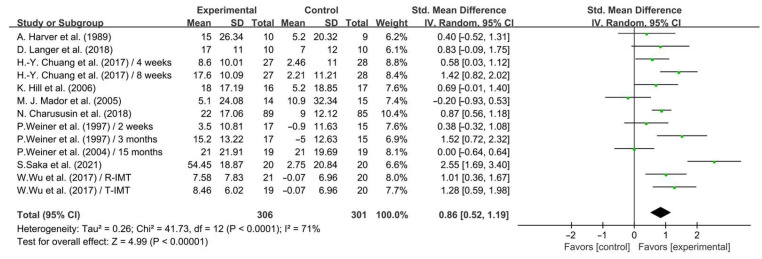
Meta-analysis results on the effects of IMT on inspiratory muscle strength in COPD patients [23,24,25,26,28,29,32,36,37,38].

As shown in Figure 2, our results showed that IMT significantly enhanced inspiratory muscle strength in COPD patients (SMD = 0.86; 95% CI: 0.52 to 1.19, *p* < 0.00001, *I*^2^ = 71%).

**Figure 3:**
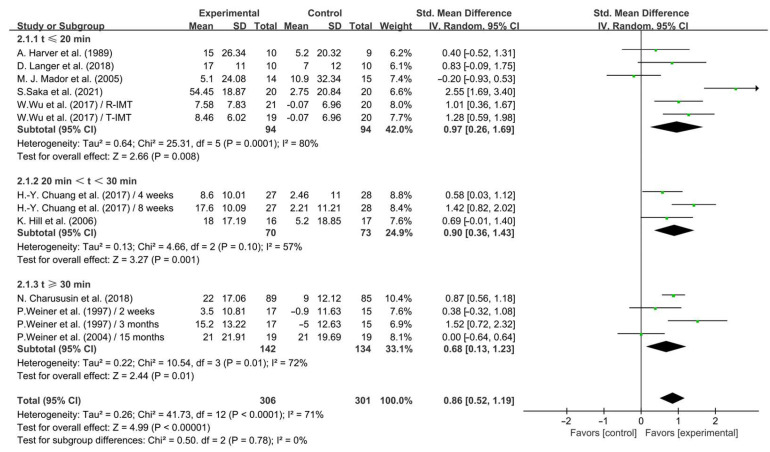
Meta-analysis results on the effects of session duration on inspiratory muscle strength in COPD patients [23,24,25,26,28,29,32,36,37,38].

Subgroup analysis showed that IMT conducted for ≤20 min (SMD = 0.97; 95% CI: 0.26 to 1.69, *p* = 0.008, *I*^2^ = 80%), between 20 and 30 min (SMD = 0.90; 95% CI: 0.36 to 1.43, *p* = 0.001, *I*^2^ = 57%), and ≥30 min (SMD = 0.68; 95% CI: 0.13 to 1.23, *p* = 0.01, *I*^2^ = 72%, Figure 3) significantly enhanced inspiratory muscle strength in COPD patients, with IMT conducted for ≤20 min having a greater effect.

**Figure 4:**
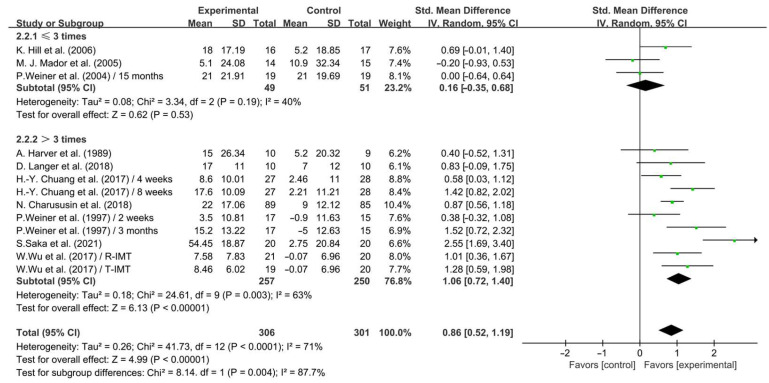
Meta-analysis results on the effects of frequency of intervention on inspiratory muscle strength in COPD patients [23,24,25,26,28,29,32,36,37,38].

In addition, when analyzing the subgroups by frequency, IMT conducted >3 times per week significantly enhanced inspiratory muscle strength in COPD patients (SMD = 1.06; 95% CI: 0.72 to 1.40, *p* < 0.00001, *I*^2^ = 63%), while IMT conducted ≤3 times per week had no significant effect on enhancing inspiratory muscle strength in COPD patients (SMD = 0.16; 95% CI: −0.35 to 0.68, *p* = 0.53, *I*^2^ = 40%, Figure 4).

**Figure 5:**
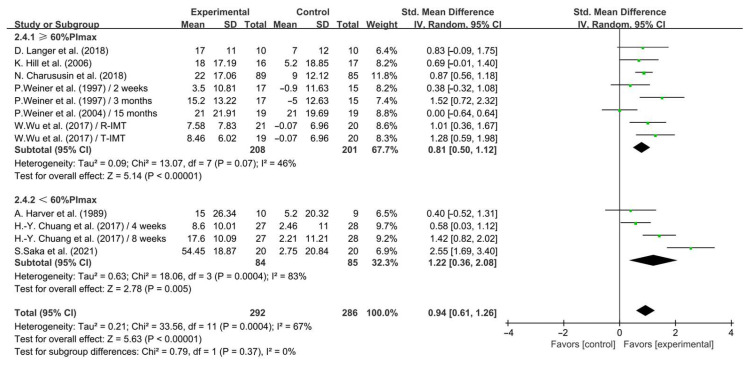
Meta-analysis results on the effects of intensity of intervention on inspiratory muscle strength in COPD patients [23,24,25,26,28,32,36,37,38].

Furthermore, when analyzing the subgroup by intensity, <60% PImax IMT (SMD = 1.22; 95% CI: 0.36 to 2.08, *p* = 0.005, *I*^2^ = 83%) and ≥60% PImax IMT (SMD = 0.81; 95% CI: 0.50 to 1.12, *p* < 0.00001, *I*^2^ = 46%, Figure 5) significantly enhanced inspiratory muscle strength in COPD patients, with <60% PImax IMT having a greater effect.

#### 3.3.2. Effects of IMT on Dyspnea in COPD Patients

**Figure 6:**
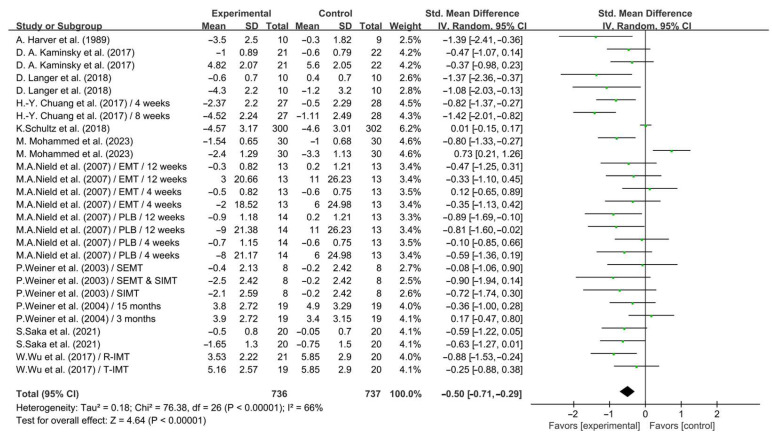
Meta-analysis results on the effects of IMT on dyspnea in COPD patients [24,25,27,28,30,31,32,33,35,36,38].

As shown in Figure 6, IMT significantly improved dyspnea in COPD patients (SMD = −0.50; 95% CI: −0.71 to −0.29, *p* < 0.00001, *I*^2^ = 66%).

**Figure 7:**
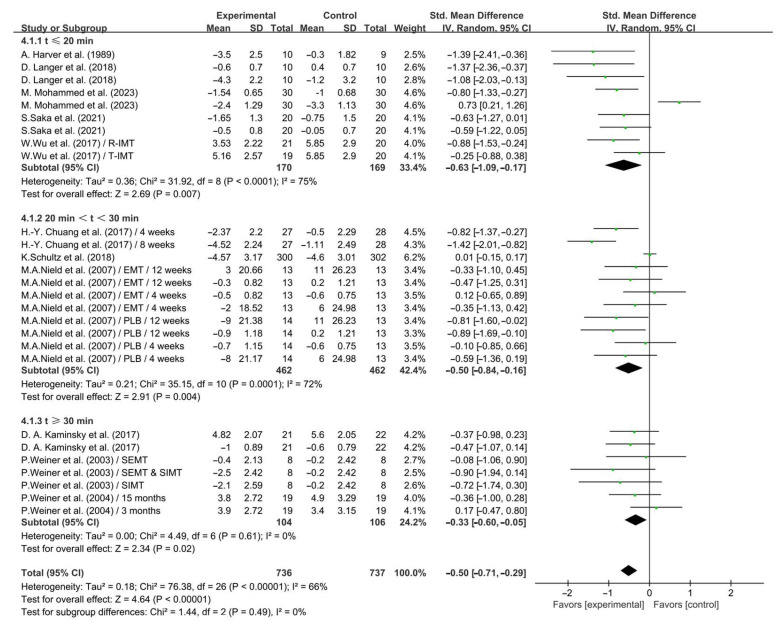
Meta-analysis results on the effects of session duration on dyspnea in COPD patients [24,25,27,28,30,31,32,33,35,36,38].

Subgroup analysis showed that IMT conducted for ≤20 min (SMD = −0.63; 95% CI: −1.09 to −0.17, *p* = 0.007, *I*^2^ = 75%), between 20 and 30 min (SMD = −0.50; 95% CI: −0.84 to −0.16, *p* = 0.004, *I*^2^ = 72%), and ≥30 min (SMD = −0.33; 95% CI: −0.60 to −0.05, *p* = 0.02, *I*^2^ = 0%, Figure 7) significantly alleviated dyspnea in COPD patients, with IMT conducted for ≤20 min having a greater effect.

**Figure 8:**
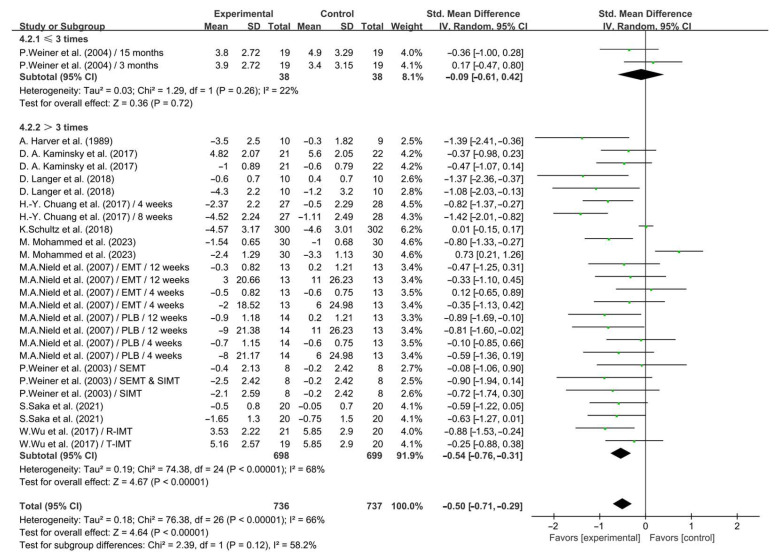
Meta-analysis results on the effects of frequency of intervention on dyspnea in COPD patients [24,25,27,28,30,31,32,33,35,36,38].

In addition, when analyzing the subgroups by frequency, IMT conducted >3 times per week significantly alleviated dyspnea in COPD patients (SMD = −0.54; 95% CI: −0.76 to −0.31, *p* < 0.00001, *I*^2^ = 68%), while IMT conducted ≤3 times per week had no significant effect on alleviating dyspnea in COPD patients (SMD = −0.09; 95% CI: −0.61 to 0.42, *p* = 0.72, *I*^2^ = 22%, Figure 8).

**Figure 9:**
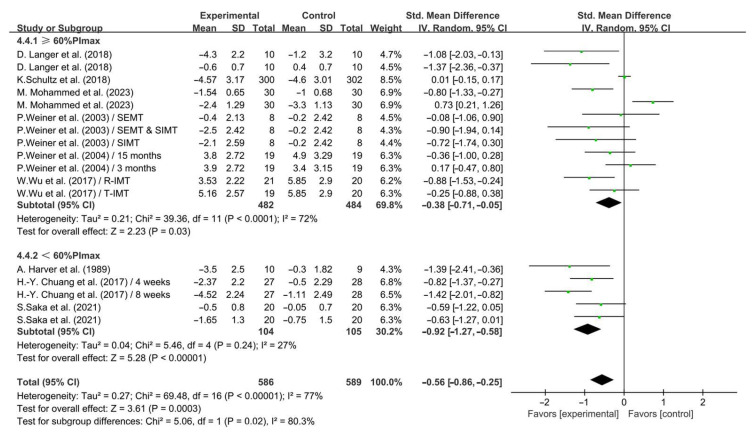
Meta-analysis results on the effects of intensity of intervention on dyspnea in COPD patients [24,25,28,30,32,33,35,36,38].

Furthermore, when analyzing the subgroups by intensity, <60% PImax IMT (SMD = −0.92; 95% CI: −1.27 to −0.58, *p* < 0.0001, *I*^2^ = 27%) and ≥60% PImax IMT (SMD = −0.38; 95% CI: −0.71 to −0.05, *p* = 0.03, *I*^2^ = 72%, Figure 9) significantly alleviated dyspnea in COPD patients, with <60% PImax IMT having a greater effect.

#### 3.3.3. Effects of IMT on QOL in COPD Patients

**Figure 10:**
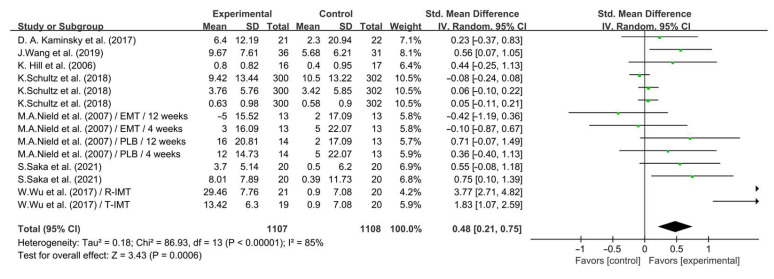
Meta-analysis results on the effects of IMT on QOL in COPD patients [26,27,31,32,33,34,38].

As shown in Figure 10, IMT significantly improved QOL in COPD patients (SMD = 0.48; 95% CI: 0.21 to 0.75, *p* = 0.0006, *I*^2^ = 85%).

**Figure 11:**
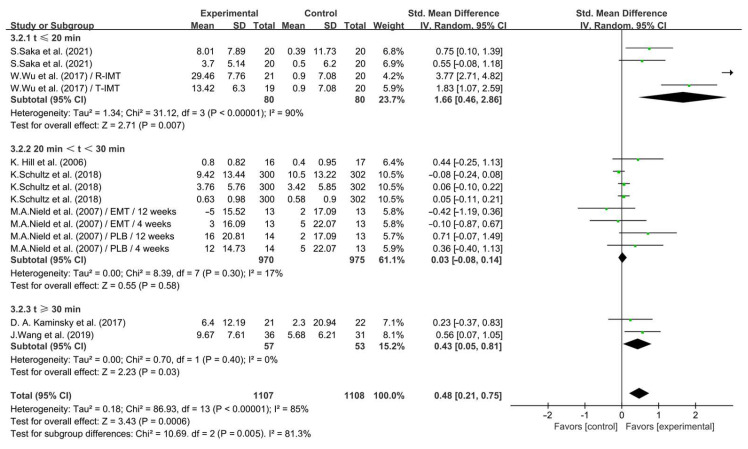
Meta-analysis results on the effects of session duration on QOL in COPD patients [26,27,31,32,33,34,38].

Subgroup analysis showed that IMT conducted for ≤20 min significantly enhanced QOL in COPD patients (SMD = 1.66; 95% CI: 0.46 to 2.86, *p* = 0.007, *I*^2^ = 90%); IMT conducted between 20 and 30 min had no significant effect on enhancing QOL in COPD patients (SMD = 0.03; 95% CI: −0.08 to 0.14, *p* = 0.58, *I*^2^ = 17%); and IMT conducted ≥30 min (SMD = 0.43; 95% CI: 0.05 to 0.81, *p* = 0.03, *I*^2^ = 0%, Figure 11) significantly enhanced QOL in COPD patients, with IMT conducted for ≤20 min having a greater effect.

**Figure 12:**
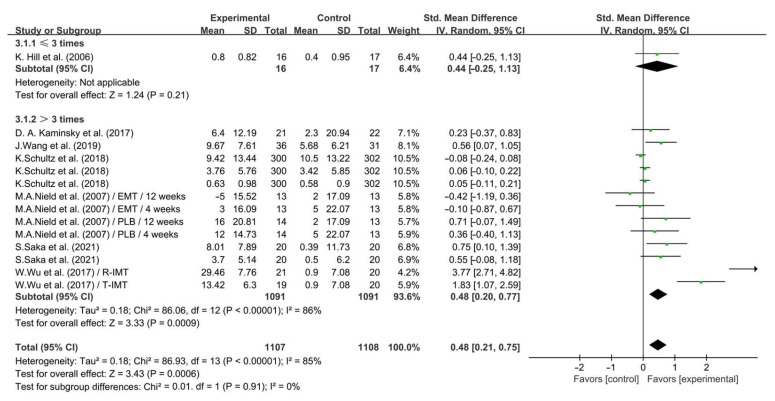
Meta-analysis results on the effects of frequency of intervention on QOL in COPD patients [26,27,31,32,33,34,38].

In addition, when analyzing the subgroups by frequency, IMT conducted >3 times per week significantly enhanced QOL in COPD (SMD = 0.48; 95% CI: 0.20 to 0.77, *p* = 0.0009, *I*^2^ = 86%), while IMT conducted ≤3 times per week had no significant effect on enhancing QOL in COPD patients (SMD = 0.44; 95% CI: −0.25 to 1.13, *p* = 0.21, Figure 12).

**Figure 13:**
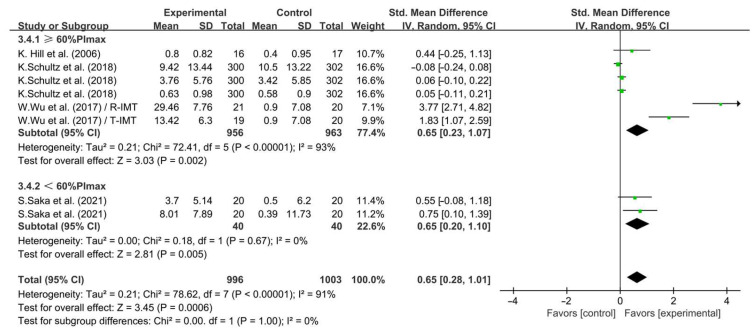
Meta-analysis results on the effects of intensity of intervention on QOL in COPD patients [26,32,33,38].

Furthermore, when analyzing the subgroups by intensity, <60% PImax IMT (SMD = 0.65; 95% CI: 0.20 to 1.10, *p* = 0.005, *I*^2^ = 0%) and ≥60% PImax IMT (SMD = 0.65; 95% CI: 0.23 to 1.07, *p* = 0.002, *I*^2^ = 93%, Figure 13) significantly enhanced QOL in COPD. However, the effect of IMT at <60% PImax and ≥60% PImax on enhancing the QOL of COPD patients was equivalent.

### 3.4. Risk of Bias

The RoB2 tool was utilized to evaluate the methodological quality of the included literature, emphasizing six domains: random bias, performance bias, detection bias, reporting bias, and other bias. As depicted in Figure S1, the overall quality of the studies was stratified into three levels: low, moderate, and high. Specifically, seven studies demonstrated a low risk of bias, four studies showed a moderate risk, and five studies were categorized as having a high risk of bias.

### 3.5. Sensitivity Analyses

Sensitivity analysis indicated that the positive effects of exercise on inspiratory muscle strength (Figure S2), dyspnea (Figure S3), and QOL (Figure S4) in COPD patients remained stable and consistent in both direction and magnitude, regardless of the exclusion of any individual study.

### 3.6. Publication Bias

To evaluate the possibility of publication bias, funnel plots were generated (Figures S3–S5). The results of Egger’s test suggested that the small-sample-size studies did not significantly impact the overall results, particularly for inspiratory muscle strength (*p* = 0.834). However, exceptions were observed for dyspnea (*p* = 0.001) and QOL (*p* = 0.013, Table S2), suggesting potential bias in these areas. In response, Dsuval and Tweedie’s trim and fill procedure was applied, and the subsequent analysis confirmed the absence of evidence for publication bias in relation to dyspnea and QOL.

## 4. Discussion

The purpose of this study was to explore the effects of IMT on inspiratory muscle strength, dyspnea, and QOL in COPD patients. A total of 16 studies were included, with the results conclusively demonstrating that IMT significantly improved inspiratory muscle strength, dyspnea, and QOL in COPD patients.

### 4.1. Effects of IMT on Inspiratory Muscle Strength in COPD Patients

We found that IMT can significantly improve inspiratory muscle strength in COPD patients. This finding is consistent with previous studies [39] showing that 12 weeks of home-based IMT significantly improved PImax. Figueiredo et al. [9] showed that inspiratory muscle strength can be notably improved through IMT alone or in combination with other interventions. In addition, Wanke et al. [40] showed that a rehabilitation program incorporating both IMT and cardiorespiratory exercise training (CET) could improve inspiratory muscle function, whereas CET alone did not produce the same effect. Furthermore, Tounsi et al. [41] demonstrated that a combination of IMT and educational exercises, performed once daily for 4–5 min over 7 days per week, led to a more pronounced improvement in inspiratory muscle strength compared to educational exercises alone. Moreover, Shahin et al. [42] revealed that, in patients with severe COPD, IMT conducted six times a week can improve inspiratory muscle strength, alleviate dyspnea, and improve prognosis regarding inspiratory function (IF).

The primary abnormalities in respiratory muscle function in COPD patients are believed to stem from mechanical defects caused by overinflation, which shorten the diaphragm fibers and force it to work on an ineffective portion of its length–tension curve [43]. Specifically, the loss of lung elastic recoil and the development of expiratory flow limitation both contribute to progressive air trapping, increased end-expiratory lung volume, and reduced inspiratory capacity [44]. A systematic review has shown that IMT is more beneficial for inspiratory muscle strength in COPD patients when the training intensity is reduced [9]. Consequently, IMT in COPD patients can improve inspiratory muscle strength by improving the contractility and relaxation of inspiratory muscle. Recent research suggests that, when combined with pulmonary rehabilitation (PR), IMT may not necessarily improve dyspnea, respiratory muscle strength, or QOL, though larger effects in participants with weak respiratory muscles and longer training durations remain to be confirmed [45]. This may be intimately tied to the intensity, frequency, and duration of respiratory muscle training.

Based on the subgroup analysis of inspiratory muscle strength, when the intervention duration of IMT is ≤20 min per session, the improvement in inspiratory muscle strength in COPD patients is optimal. This finding concurs with a previous study [46], which demonstrated a significant enhancement in PImax in COPD patients who underwent 15 min of home-based IMT per session. This may stem from the fact that excessively brief intervention times may lack efficacy, whereas excessively long intervention times can induce respiratory muscle fatigue, subsequently diminishing inspiratory muscle improvement.

In addition, our subgroup analysis revealed that when IMT was conducted >3 times per week, the improvement in inspiratory muscle strength was most pronounced, which was consistent with previous studies. Weiner et al. [36] demonstrated that IMT leads to an improvement in inspiratory muscle strength; specifically, PImax significantly increased in COPD patients who underwent IMT six times per week. Battaglia et al. [36], on the other hand, reported on the combined use of a novel expiratory device alongside a previously evaluated inspiratory device, which enhanced inspiratory muscle strength in COPD patients receiving IMT seven times per week IMT. Furthermore, the ACSM recommends that COPD patients engage in 8 to 10 sessions per week of activities aimed at maintaining or enhancing muscle strength and endurance to maximize strength gains. Moreover, our findings indicated that IMT with an intensity of <60% PImax was more efficacious in improving inspiratory muscle strength in COPD patients, consistent with previous studies. Saka et al. [32] demonstrated that in the IMT group, intensity was initially set at 30% PImax and subsequently adjusted based on weekly PImax values. Following IMT, a statistically significant improvement in PImax was observed. Xu et al. [32], on the other hand, reported that IMT with an intensity of 45% PImax alone is an effective method for improving inspiratory muscle strength. In conclusion, IMT sessions lasting ≤ 20 min, conducted > 3 times per week, and at an intensity of <60% PImax are more beneficial for enhancing inspiratory muscle strength in COPD patients.

### 4.2. Effects of IMT on Dyspnea in COPD Patients

Our results demonstrated that IMT significantly alleviated dyspnea in COPD patients, which is consistent with previous studies. For example, Beaumont et al. [47] showed that threshold IMT can significantly improve dyspnea in COPD patients. In addition, Battaglia et al. [48] reported that combining home exercise using expiratory and inspiratory devices can significantly improve dyspnea in patients with mild to severe COPD. Furthermore, Shahin et al. [42] presented results indicating that IMT not only improves the perception of dyspnea but also improves IF outcomes in patients with severe COPD. Previous studies have underscored the pivotal role of respiratory muscle weakness in the development of dyspnea. Therefore, dyspnea is frequently used as a prognostic indicator in COPD studies [49]. Dyspnea in COPD patients arises from an increased respiratory rate due to pulmonary hyperinflation, leading to rapid and shallow breathing. This breathing pattern decreases the gas exchange rate and an exacerbates respiratory muscle fatigue. During exercise, COPD patients experience increased lactic acidosis and are unable to meet their ventilation demands. IMT fortifies muscle strength and endurance by exercising the inspiratory muscles, mainly the diaphragm, and augments patients’ ventilation capacity, thereby mitigating dyspnea [50]. It alleviates dyspnea in COPD patients by enhancing inspiratory muscle strength. Nevertheless, some researchers remain skeptical regarding the dyspnea-improving effects of IMT. Figueiredo et al. [9] showed that isolated IMT did not alleviate dyspnea, and the presence of inspiratory muscle weakness did not alter this outcome. In addition, Koch et al. [51] reported that a small number of subjects exhibited a placebo effect following independent IMT, and the effectiveness of independent IMT did not significantly differ from that of the control group. These studies suggest that the effectiveness of IMT in improving dyspnea in COPD patients is influenced by factors such as the patient’s disease status, intervention type, intensity, and frequency, and a unified conclusion has yet to be reached.

Based on the subgroup analysis of dyspnea, IMT conducted for ≤20 min yielded the most pronounced improvement in dyspnea in COPD patients, which is consistent with previous studies. Battaglia et al. [32] reported that COPD patients progressed with IMT for 15 min per session. The results showed that COPD patients’ dyspnea significantly alleviated. Buran et al. [52] demonstrated that after 15 min of IMT combined with manual therapy (MT), COPD patients’ dyspnea significantly decreased. In addition, IMT conducted >3 times per week was more effective in alleviating dyspnea in COPD patients, which aligns with previous studies. Weiner et al. [36] showed that IMT resulted in significant improvement in dyspnea when COPD patients received IMT six times per week. Battaglia et al. [48] reported that after COPD patients performed IMT seven times per week, dyspnea values were significantly improved. Furthermore, IMT at an intensity of <60% PImax was more effective in alleviating dyspnea in COPD patients, which is in agreement with the findings of Saka et al. [32], who showed that a 30% PImax IMT load can facilitate the alleviation of dyspnea.

### 4.3. Effects of IMT on QOL in COPD Patients

Decreased QOL serves as a predictor for mortality and re-hospitalization in COPD patients. Research has established that both inspiratory and expiratory muscle training can improve the QOL in COPD patients. Notably, the improvement in QOL observed in IMT groups was significantly greater than that in control groups, likely due to the more substantial reduction in dyspnea achieved through IMT. Addressing dyspnea through respiratory muscle training contributes to improving QOL [53]. COPD is characterized by its progressive and insidious nature, with lung function often declining by 50% before symptoms manifest. A sudden acute exacerbation of symptoms significantly impact patients’ QOL and treatment costs. As a standalone treatment, IMT can mitigate dyspnea and improve exercise tolerance, thereby improving the QOL of COPD patients by reducing dyspnea and slowing disease progression [50].

In this study, we have discovered that IMT improves the QOL in COPD patients by alleviating dyspnea, which is consistent with previous studies. Abedi et al. [50] has shown that a combination of IMT and aerobic exercise has a marked effect on improving QOL. In addition, Sánchez et al. [54] demonstrated that 6 days of weekly, targeted IMT significantly contributed to improving QOL. Furthermore, Ozsoy et al. [49] revealed that both 4 weeks of basic IMT and 4 weeks of functional IMT (aiming to enhance all muscle functions for greater benefits) notably improved the QOL in elderly COPD patients.

Concurrently, some researchers have expressed doubts about the extent of IMT’s benefits on QOL in COPD patients. While IMT can significantly improve respiratory muscle function, its impact on lung function, clinical outcomes, and QOL remains unsubstantiated by robust scientific evidence. Polkey et al. [55] suggested that the decrease in diaphragmic pressure observed in COPD patients is primarily attributed to hyperinflation, which they believe is unlikely to be ameliorated by IMT. They further posited that the link between the decline in inspiratory pressure caused by hyperinflation and adverse outcomes might be an epiphenomenon. O’Brien et al. [12] found that IMT significantly improved inspiratory muscle strength and endurance, yet the benefits in terms of sports performance and QOL were not evident.

According to the subgroup analysis of QOL, when the intervention duration of IMT is ≤20 min per session, the improvement in QOL in COPD patients is optimal, which aligns with previous studies. Beaumont et al. [47] reported that IMT for 15 min per session combined with a pulmonary rehabilitation program (PRP) is an effective way to improve the QOL of COPD patients. Buran et al. [52] revealed that after 15 min of IMT combined with MT, a comparison between COPD patients’ QOL and the control group showed significant different changes in the total and all subscale scores of QOL. In addition, IMT conducted > 3 times per week was more effective in improving QOL, which is consistent with previous studies. Xu et al. [53] showed that IMT at a frequency of seven times per week can improve symptoms of depression, anxiety, insomnia, and QOL in COPD patients. Sánchez et al. [54] demonstrated that targeted IMT six times per week improves QOL in COPD patients. However, an intervention frequency of ≤3 times per week did not affect the improvement of QOL. Since only one study with ≤3 times per week was included in this subgroup, further studies are needed to verify this result in the future. Furthermore, our subgroup analysis indicated that both intervention intensities of <60% PImax and ≥60% PImax were effective in improving QOL, which is in line with previous studies. Xu et al. [53] showed that 45% PImax IMT significantly improved QOL compared to sham training. Beaumont et al. [47] reported that in severe and very severe COPD patients, 60% PImax IMT performed during a PRP is associated with an improvement in QOL.

### 4.4. Strengths and Limitations of This Study

There are some potential limitations to this meta-analysis. Firstly, all the included studies are RCTs of IMT interventions, which cannot be fully blinded, potentially introducing a degree of bias in the quality evaluation due to subjective factors. Secondly, while the included studies did not explicitly identify intervention-related adverse events, it is uncertain whether the investigators comprehensively documented all possible adverse events. Finally, the subgroup analysis did not encompass the monitoring of intervention measures and rest periods during exercise. Consequently, there may be a necessity for future research endeavors featuring larger sample populations and enhanced methodological rigor to augment and validate our current findings.

## 5. Conclusions

IMT is an effective approach to improving inspiratory muscle strength, alleviating dyspnea, and enhancing QOL in COPD patients. Conducting IMT for ≤20 min and >3 times per week is the optimal way to improve inspiratory muscle strength, dyspnea, and QOL, whereas IMT performed at an intensity of <60% PImax is more effective in improving inspiratory muscle strength and dyspnea in COPD patients. Since the effect of IMT at <60% PImax and ≥60% PImax on enhancing the QOL of COPD patients is equivalent, this meta-analysis provides clinicians with evidence to recommend that COPD patients engage in IMT at <60% PImax for more than three times per week, with each session lasting no more than 20 min, to improving inspiratory muscle strength, dyspnea, and QOL.

## Data Availability

All data generated or analyzed during this study are included in the article/Supplementary Material.

## Supplementary Materials

The following supporting information can be downloaded at https://www.mdpi.com/article/10.3390/life14111470/s1, Figure S1: Results of Cochrane risk-of-bias tool; Figure S2: Sensitivity analysis results on inspiratory muscle strength; Figure S3: Sensitivity analysis results on dyspnea; Figure S4: Sensitivity analysis results on QOL; Figure S5: Funnel plot of inspiratory muscle strength; Figure S6: Funnel plot of inspiratory dyspnea; Figure S7: Funnel plot of inspiratory QOL; Table S1: Characteristics of the studies included in this meta-analysis; Table S2: Results of Egger’s test.
