# Supplementary material for: Effects of Inspiratory Muscle Training in People with Chronic Obstructive Pulmonary Disease: A Systematic Review and Meta-Analysis
Source: Life (Basel). 2024 Nov 12;14(11):1470. doi: 10.3390/life14111470 (PMC11595718; doi:10.3390/life14111470)
Supplement: Supplementary file 1 [file life-14-01470-s001.zip › life-3232051-supplementary.pdf]

Supplemental material

Effects of inspiratory muscle training in people with chronic obstructive pulmonary disease: a systematic review and meta-analysis

Figure S1 Results of Cochrane risk of bias tool....................................... 2
Figure S2 Sensitivity analyses results of inspiratory muscle strength...... 3
Figure S3 Sensitivity analyses results of dyspnea.................................... 4
Figure S4 Sensitivity analyses results of QOL... 5
Figure S5 Funnel plot of inspiratory muscle strength............................... 6
Figure S6 Funnel plot of inspiratory dyspnea... 7
Figure S7 Funnel plot of inspiratory QOL.. 8
Table S1 Characteristics of the studies included in this meta-analysis...... 9
Table S2 Results of Egger's test... 14

Figure S1 Results of Cochrane risk of bias tool

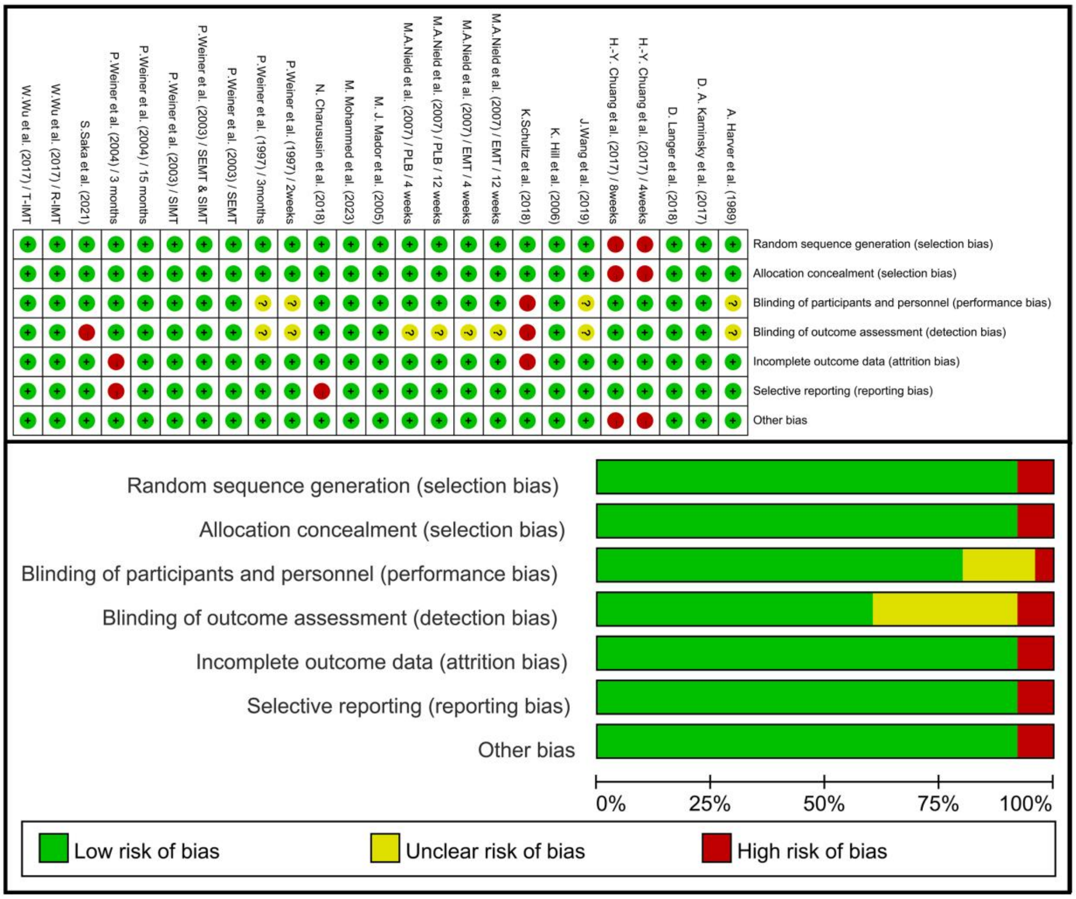

Figure S2 Sensitivity analyses results of inspiratory muscle strength

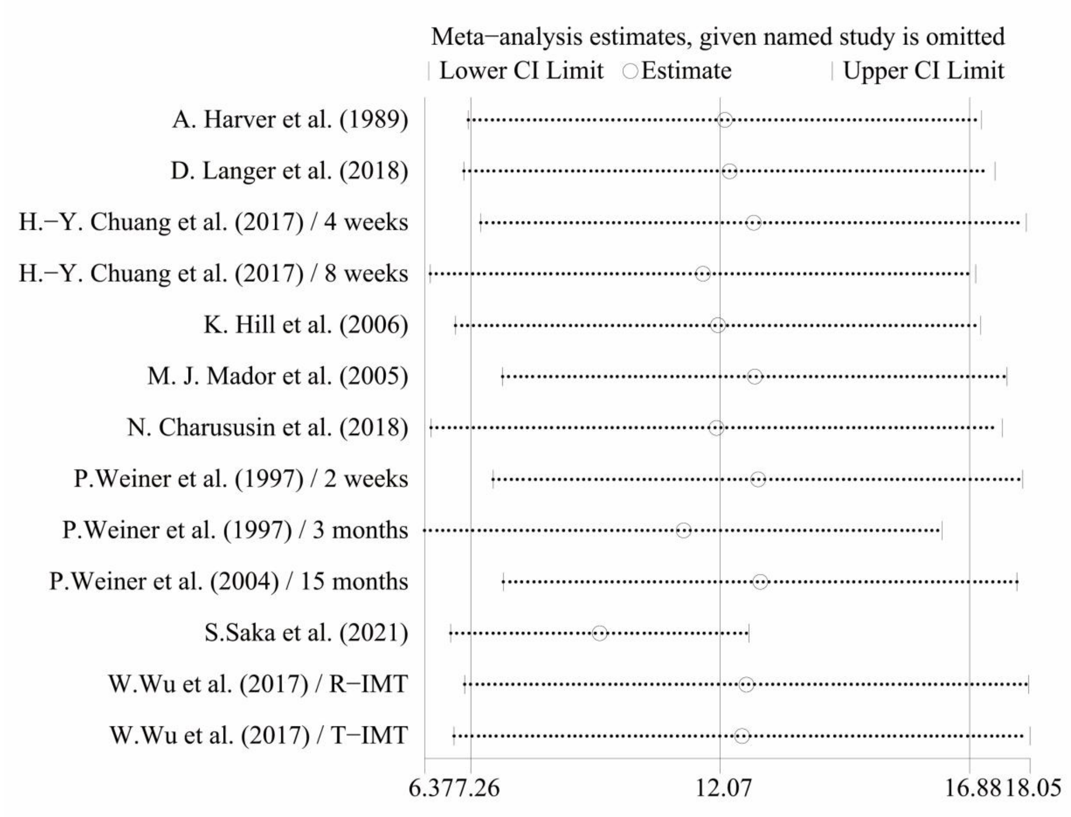

Figure S3 Sensitivity analyses results of dyspnea

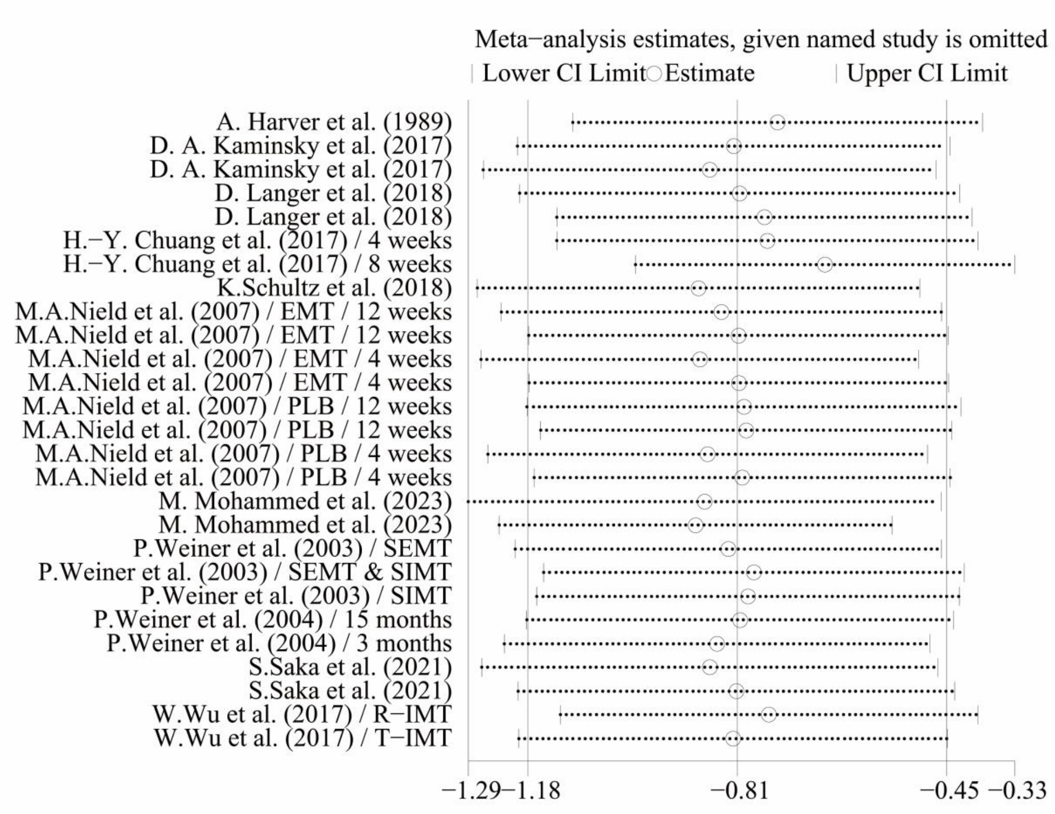

Figure S4 Sensitivity analyses results of QOL

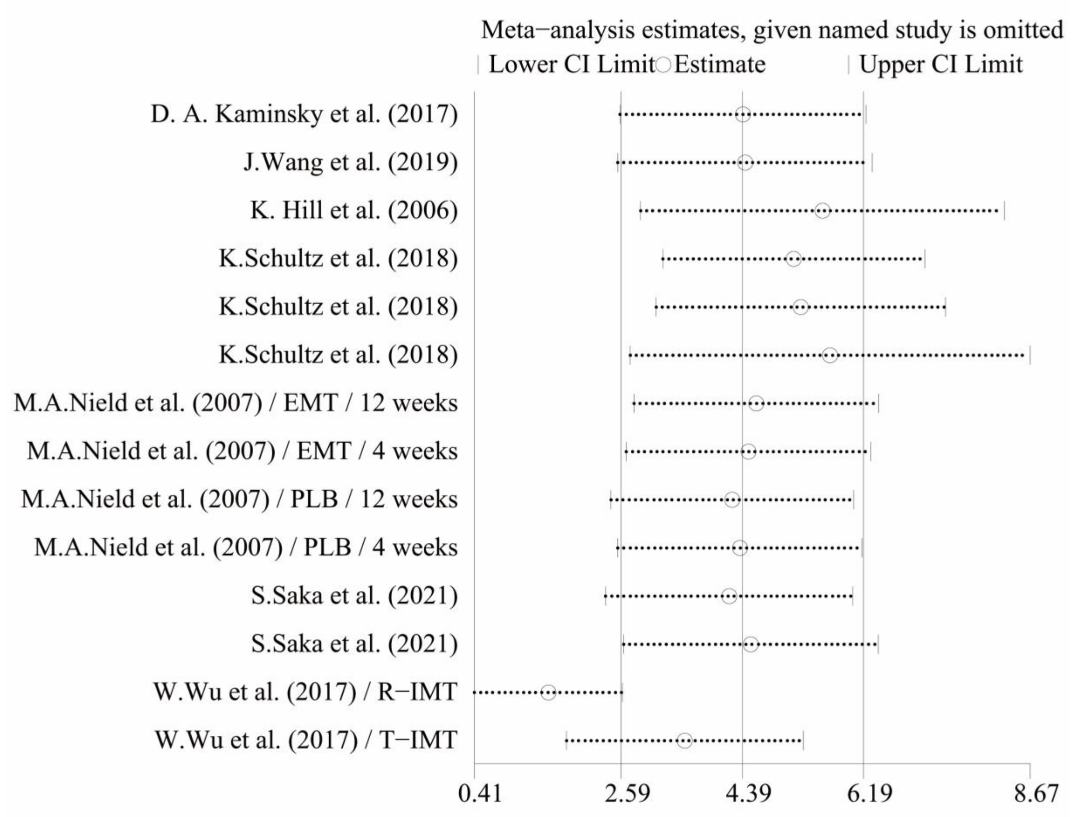

Figure S5 Funnel plot of inspiratory muscle strength

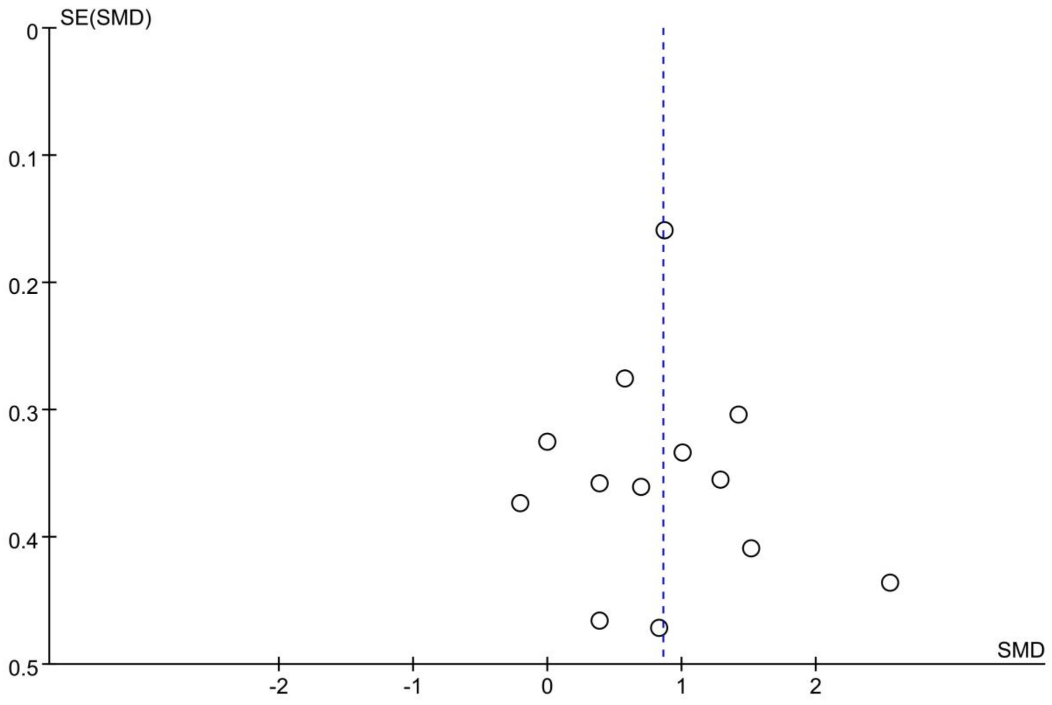

Figure S6 Funnel plot of dyspnea

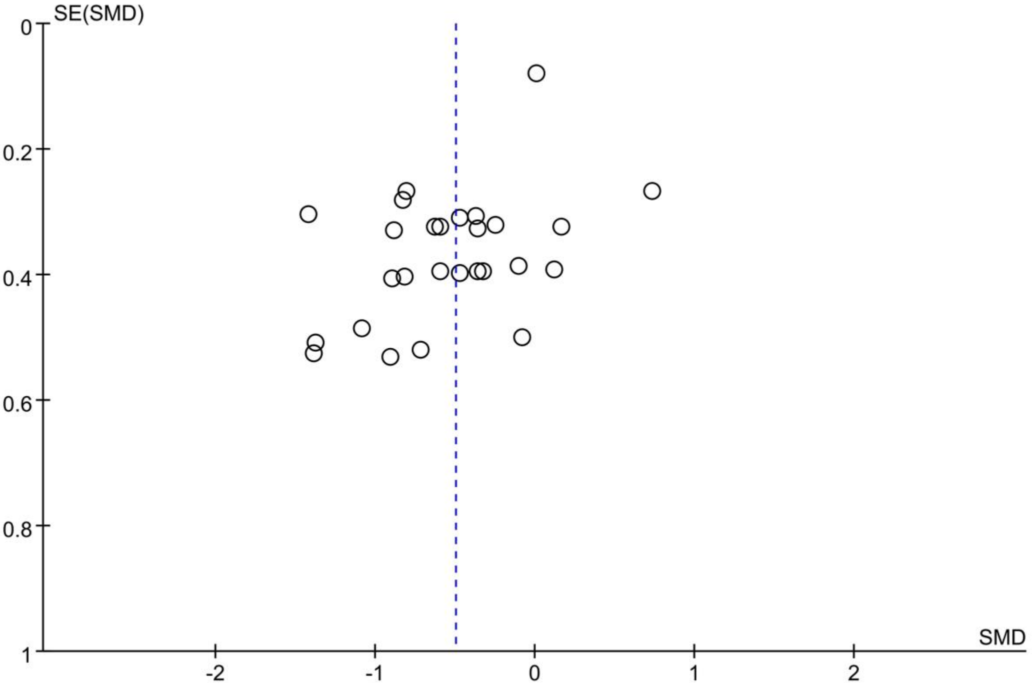

Figure S7 Funnel plot of QOL

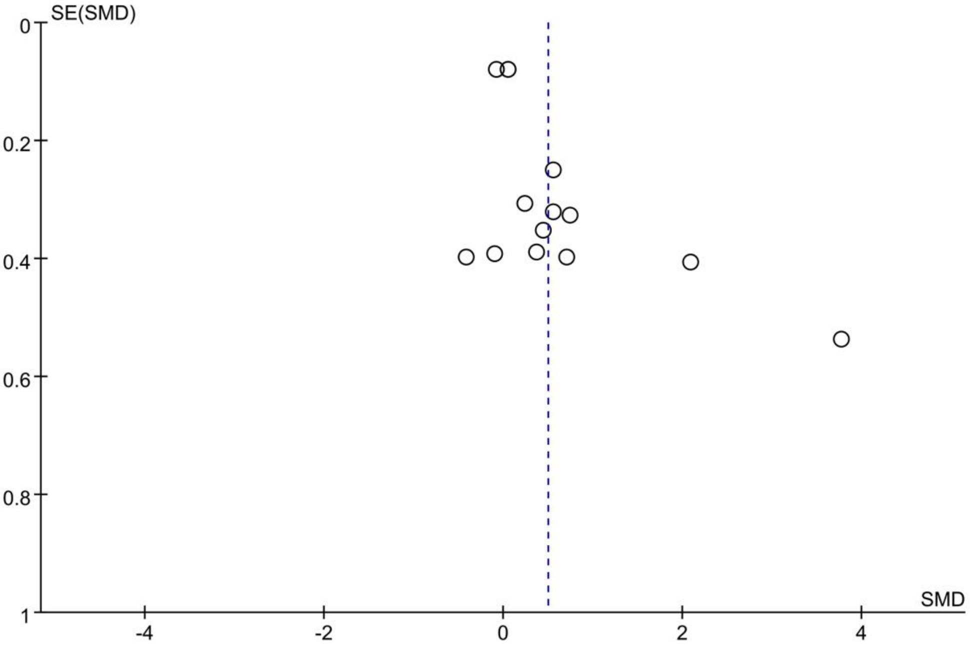

Table S1 Characteristics of the studies included in this meta-analysis

Study	Sample size	Age (y)	Stage of disease	Intervention	Setting device	Characteristics of intervention	Outcome measures
N. Charususin (2018)	IG = 89<br>CG = 85	IG: 66 (8)<br>CG: 65 (7)	Total: GOLD Ⅲ	IG: threshold IMT<br>CG: usual care	POWERbreatheKHP2	60 min/session, 47 (2)%-84 (4)% PImax, 3-5 sessions per week, for 12 weeks	PImax
H.-Y. Chuang (2017)	IG = 27<br>CG = 28	IG: 66.22 (12.76)<br>CG: 66.04 (10.99)	Total: GOLD Ⅲ	IG: threshold IMT<br>CG: usual care	Adjustable pressure threshold loading device	21-30 min/session, 15-40 cmH_2O pressure load, 5 sessions per week, for 8 weeks	PImax, 6MWT, BDI/TDI, QOL
A. Harver (1989)	IG = 10<br>CG = 9	Total: 48-76	Total: GOLD Ⅱ-Ⅳ	IG: increasing threshold IMT<br>CG: constant threshold IMT	PFLEX muscle trainer	15 min/session, 5-35 cmH_2O pressure load,<br>2 sessions each day, for 8 weeks	PImax, BDI/TDI
K. Hill (2006)	IG = 16<br>CG = 17	IG: 69.4 (7.2)<br>CG: 66.6	Total: GOLD Ⅲ	IG: high level threshold IMT	Threshold IMT	21 min/session, increased to 101% PImax (IG);	PImax, CRDQ

		(9.8)		CG: constant threshold IMT		10% PImax (CG), 3 sessions per week, for 8 weeks	
D. A. Kaminsky (2017)	IG = 21<br>CG = 22	IG: 68 (7)<br>CG: 68 (9)	Total: GOLD Ⅱ - Ⅳ	IG: pranayama training<br>CG: usual care	NR	60 min/session, 30 min pranayama 30 min education (IG); 60 min education (CG), 7 day per week, for 12 weeks	mMRC, BDI/TDI, SGRQ
D. Langer (2018)	IG = 10<br>CG = 10	IG: 73 (4)<br>CG: 67 (8)	Total: GOLD Ⅲ	IG: increasing threshold IMT<br>CG: constant threshold IMT	POWERbreathe®KH2	4-5 min/session, 40% PImax to highest to lerable intensity (IG); 10% PImax (CG), 2 (IG), 3 (CG), 7 day per week, for 8 weeks	mMRC, BDI/TDI, PImax
M. J. Mador (2005)	IG = 14<br>CG = 15	IG: 70.9 (7.48)<br>CG: 69.7 (7.75)	Total: GOLD Ⅲ	IG: endurance training<br>CG: ET & hyperpnea training	Rebreathing bag, noseclips	20 min/session, at least 50% Wmax and at least 1.1-2 mile/h (IG); hyperpnea 15-20 min target ventilation increase by 5% 10% (CG), 24 sessions for 8 weeks	PImax
M. Mohammed (2023)	IG = 30<br>CG = 30	IG: 63.333 (7.8)	Total: GOLD Ⅱ - Ⅳ	IG: threshold IMT<br>CG: DB + PLB	The classic POWER breathe device	4-5 min/session, 30%-60% PImax, 2 sessions per day, 7 day per week,	mMRC, Borg scale

		CG: 60.4 (5.3)		breathing		for 12 weeks	
M. A. Nield (2007)	IG-PLB = 14<br>IG-EMT = 13<br>CG = 13	IG-PLB: 62 (12)<br>IG-EMT: 63 (5)<br>CG: 69 (8)	Total: GOLD Ⅲ	IG-PLB: pursed-lips breathing<br>IG-EMT: EMT<br>CG: usual care	IG-PLB: Nellcor N-395, Nonin 9500<br>IG-EMT: ThresholdiPEP	10-25 min/session, 10% PEmax, 7 day per week, for 4 weeks	Borg scale, SOBQ, SF-36
S. Saka (2021)	IG = 20<br>CG = 20	IG: 62.3 (7.43)<br>CG: 62.1 (7.76)	Total: GOLD Ⅲ	IG: increasing threshold IMT<br>CG: constant threshold IMT	Threshold IMT® Philips Respironics	15 min/session, at least 30% PImax (IG); 10% PImax (CG), 2 sessions per day, 5 days per week,for 8 weeks	mMRC, Borg, PImax, SGRQ, CAT
K. Schultz (2018)	IG = 300<br>CG = 302	IG: 57.7 (8.2)<br>CG: 57.9 (6.6)	IG: GOLD Ⅱ<br>CG: GOLD Ⅲ	IG: High-intensity intervals<br>CG: sham IMT	POWERbreathe Medic	21 min/session, 30%-60% PImax (IG); < 1 cmH_2O (CG), 7 sessions per week, for 3 weeks	SGRQ, CAT, CCQ, BDI/TDI
J. Wang (2019)	IG = 36	IG: 63.82 (19.64)	Total: GOLD Ⅱ	IG: exspiratory muscle training	SpiroTiger	30 min/session, breathing frequency: 10-20 times/min, 5	CAT

	CG = 31	CG: 61.92 (17.92)		CG: usual care		sessions per week, for 24 weeks	
P. Weiner (2003)	IG-SEMT = 8<br>IG-SIMT = 8<br>IG-SEMT + SIMT = 8<br>CG = 8	IG-SEMT: 65.4 (9.33)<br>IG-SIMT: 63.1 (8.77)<br>IG-SEMT + SIMT: 62.7 (8.49)<br>CG: 61.8 (9.05)	Total: GOLD Ⅲ	IG-SEMT: SEMT<br>IG-SIMT: SIMT<br>IG-SEMT + SIMT: SEMT + SIMT<br>CG: low pressure load training	Threshold Inspiratory Muscle Trainer	60 min/session, 15%-60% PImax (SEMT/SIMT/SEMTSIMT); 7 cmH_2O (CG), 6 sessions per week, for 12 weeks	BDI/TDI
P. Weiner (2004)	IG = 19<br>CG = 19	IG: 66.3 (14.82)<br>CG: 64.9 (13.95)	Total: GOLD Ⅲ	IG: increasing threshold IMT<br>CG: constant threshold IMT	ThresholdTM Inspiratory Muscle Trainer	30 min/session, stage1: 10%, 60% PImax (IG&CG), 6 sessions per week, for 12 weeks, stage 2: 60% PImax (IG); 7 cmH_20 (CG), 3 sessions per week, for 12 months	BDI/TDI, PImax
P. Weiner (1997)	IG = 17	Total: 61.5	GOLD Ⅳ	IG: SIMT	THRESHOLD	60 min/session,15-60% PImax, 6	PImax

	CG = 15	(9.62)		CG: usual care	inspiratory muscle trainer, DHD Coach	sessions per week, for 12 weeks	
W. Wu (2017)	IG-R-IMT = 21<br>IG-T-IMT = 19<br>CG = 20	IG-R-IMT: 62.24 (7.36)<br>IG-T-IMT: 59.74 (6.14)<br>CG: 60.30 (6.55)	Total: GOLD Ⅱ	IG-R-IMT: resistive IMT<br>IG-T-IMT: threshold IMT<br>CG: usual care	IG-R-IMT: PFLEX<br>IG-T-IMT: Threshold Inspiration Muscle Trainer	15 min/session, 60% PImax, 2 sessions per day, daily practice, for 8 weeks	BDI/ TDI, CRDQ

Abbreviations: COPD, chronic obstructive pulmonary disease; IG, intervention group; CG, control group; NR, not reported; PLB, pursed-lip breathing; EMT, expiratory muscle training; SEMT, specific expiratory muscle training; SIMT, specific inspiratory muscle training; R-IMT, resistive inspiratory muscle training; T-IMT, threshold inspiratory muscle training; GOLD, global initiative for chronic obstructive lung disease; ET, endurance training; PImax, maximal inspiratory muscle pressure; 6MWT, 6-minute walk test; BDI/TDI, baseline dyspnea index/transition dyspnea index; QOL, quality of life; CRDQ, chronic respiratory disease questionnaire; mMRC, modified medical research council; SGRQ, St George's Respiratory Questionnaire; CAT, chronic airway test; CCQ, clinical COPD questionnaire.

Table S2 Results of Egger's test

Index	Std_EFF	Coef.	Std. Err.	t	P ＞ |t|	95% CI
Inspiratory muscle strength	slope	0.7328232	0.5222959	1.4	0.188	-0.4167424, 1.882389
	bias	0.3596956	1.671405	0.22	0.834	-3.319043, 4.038434
Dyspnea	slope	0.1651209	0.1445491	1.14	0.264	-0.1325836, 0.4628254
	bias	-1.899655	0.5228655	-3.63	0.001	-2.976517, -0.8227939
QOL	slope	-0.2075283	0.1404988	-1.48	0.165	-0.5136489, 0.0985922
	bias	2.556092	0.8730976	2.93	0.013	0.653776, 4.458409

Abbreviations: QOL, quality of life; Coef., coefficient; Std. Err., standard error; t, t-test statistic; CI, Confidence Interval.
